# Impaired calcium handling mechanisms in atrial trabeculae of diabetic patients

**DOI:** 10.14814/phy2.15599

**Published:** 2023-02-07

**Authors:** Timothy L. M. Jones, Sarbjot Kaur, Nicholas Kang, Peter N. Ruygrok, Marie‐Louise Ward

**Affiliations:** ^1^ Department of Physiology University of Auckland Auckland New Zealand; ^2^ Greenlane Cardiothoracic Surgical Unit Auckland City Hospital Auckland New Zealand; ^3^ Department of Cardiology Auckland City Hospital Auckland New Zealand

**Keywords:** calcium handling, diabetes, human right atrial trabeculae, myofilament sensitivity

## Abstract

The aim of this study was to investigate cardiomyocyte Ca^2+^ handling and contractile function in freshly excised human atrial tissue from diabetic and non‐diabetic patients undergoing routine surgery. Multicellular trabeculae (283 ± 20 μm in diameter) were dissected from the endocardial surface of freshly obtained right atrial appendage samples from consenting surgical patients. Trabeculae were mounted in a force transducer at optimal length, electrically stimulated to contract, and loaded with fura‐2/AM for intracellular Ca^2+^ measurements. The response to stimulation frequencies encompassing the physiological range was recorded at 37°C. Myofilament Ca^2+^ sensitivity was assessed from phase plots and high potassium contractures of force against [Ca^2+^]_i_. Trabeculae from diabetic patients (*n* = 12) had increased diastolic (resting) [Ca^2+^]_i_ (*p* = 0.03) and reduced Ca^2+^ transient amplitude (*p* = 0.04) when compared to non‐diabetic patients (*n* = 11), with no difference in the Ca^2+^ transient time course. Diastolic stress was increased (*p* = 0.008) in trabeculae from diabetic patients, and peak developed stress decreased (*p* ≤ 0.001), which were not accounted for by reduction in the cardiomyocyte, or contractile protein, content of trabeculae. Trabeculae from diabetic patients also displayed diminished myofilament Ca^2+^ sensitivity (*p* = 0.018) compared to non‐diabetic patients. Our data provides evidence of impaired calcium handling during excitation‐contraction coupling with resulting contractile dysfunction in atrial tissue from patients with type 2 diabetes in comparison to the non‐diabetic. This highlights the importance of targeting cardiomyocyte Ca^2+^ homeostasis in developing more effective treatment options for diabetic heart disease in the future.

## Graphical abstract

The aim of this study was to investigate cardiomyocyte calcium and contractile function in freshly excised human atrial tissue from consenting diabetic and non‐diabetic patients. Trabeculae from diabetic patients (*n* = 12) had increased diastolic (resting) calcium and reduced calcium transient amplitude compared to trabeculae from non‐diabetic patients (*n* = 11), with no difference in the transient time course. Diastolic stress was increased in trabeculae from diabetic patients, and peak developed stress decreased, which were not accounted for by reduction in the cardiomyocyte, or contractile protein, content of trabeculae. Trabeculae from diabetic patients also displayed diminished myofilament calcium sensitivity. Our data provides evidence of impaired calcium handling during excitation‐contraction coupling with resulting contractile dysfunction in atrial tissue from patients with type 2 diabetes in comparison to the non‐diabetic.

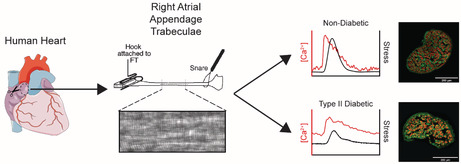

## 1 INTRODUCTION

Cardiovascular disease is the leading cause of death for patients with type 2 diabetes (Zimmet et al., 2001). While a significant proportion of these deaths is associated with comorbidities of diabetes (such as coronary artery disease, hypertension or hyperlipidemia), there is also evidence of contractile dysfunction of the heart muscle itself (Lamberts et al., 2014; Palmieri et al., 2001; Rubler et al., 1972). Despite the extremely high prevalence of type 2 diabetes in the adult population (Saeedi et al., 2019; World Health Organization, 2016), relatively little is known about the mechanisms that contribute to the cardiac dysfunction observed in human patients with this disease.

The critical importance of intracellular calcium (Ca^2+^) cycling in cardiomyocytes has been well established, due to its direct influence on the contractility of the heart (Bers, 2002; Eisner et al., 2017). Disrupted cardiomyocyte Ca^2+^ handling in diabetes has previously been reported (Allo et al., 1991; Belke & Dillmann, 2004; Pierce & Russell, 1997). Consequently, there is a dearth of information on the impact of type 2 diabetes on human cardiomyocyte function, particularly in the early stages of the disease progression. Cardiac tissue from patients with naturally derived type 2 diabetes and their non‐diabetic controls must be studied in order to obtain a more accurate understanding of the pathophysiology of diabetic heart disease.

Therefore the aims of this study were: (i) to compare the contractility of cardiac tissue obtained from patients with type 2 diabetes compared to patients without diabetes, (ii) to investigate excitation‐contraction coupling by simultaneously measuring intracellular Ca^2+^ and contractile force, and (iii) to ascertain the relative contributions of cardiomyocyte excitation–contraction coupling and cardiac muscle composition to overall muscle function. In order to accomplish our aims, we obtained freshly excised right atrial appendage (RAA) tissue samples from consenting surgical patients, with and without type 2 diabetes. Simultaneous measurements of intracellular Ca^2+^ and contractile force were made in endocardial trabeculae micro‐dissected from samples of RAA tissue. By directly measuring intracellular Ca^2+^ and contractile force human cardiac tissue from patients with naturally acquired type 2 diabetes, we aimed to provide new knowledge on the mechanisms that underlie human diabetic heart disease.

## 2 METHODS

For detailed methods, see Material S1.

### 2.1 Ethics approval and consent to participate

Informed consent was obtained from patients prior to routine coronary artery bypass grafting surgery for a small sample of RAA tissue to be obtained for our research study. The consenting process conformed to the principles outlined in the Declaration of Helsinki and was approved by the Human and Disability Ethics Committee of New Zealand (HDEC 17/STH/61) and the Auckland District Health Board Research Review Committee (A+ 7593).

### 2.2 Human RAA tissue samples

A small section (approximately 0.5 × 2 cm) of RAA tissue was obtained from consenting patients undergoing routine coronary artery bypass grafting surgery at Auckland Hospital. Tissue was excised from the RAA incision site prior to cannulation, and immediately transferred to 100 ml of Krebs–Henseleit (KH, see composition below) buffer oxygenated with 95% O_2_, 5% CO_2_, containing 0.25 mM CaCl_2_ and 20 mM 2,3‐butanedione 2‐monoxime (BDM) to minimize energy expenditure (Kirton et al., 2005; Mulieri et al., 1989). The sample was then rapidly transferred to our laboratory at the University of Auckland within 7 ± 1 min (mean ± standard error of the mean [SEM]) and super‐fusion with fresh buffer solution commenced.

Clinical notes were obtained and de‐identified patients retrospectively classified as diabetic (T2D) if their glycated hemoglobin (HbA_1c_) was 40 mmol/mol or higher, and non‐diabetic (ND) as per their clinical notes (HbA_1c_ was <40 mmol/mol).

### 2.3 Microdissection of trabeculae

The tissue was pinned out in a dissection dish and continuously super‐fused with fresh buffer solution. When presenting, an unbranched trabecula 2–4 mm long and less than 300 μm in diameter was micro‐dissected from the endocardial surface of the RAA and mounted in a temperature controlled muscle chamber (Aurora Scientific), on the stage of an inverted microscope (Nikon Eclipse TE2000‐U). Trabeculae were then continuously super‐fused with oxygenated KH buffer solutions throughout experimentation. The tissue block at one end of the trabecula was held in a wire basket extending from the beam of a force transducer (AE801, Kronex Technologies) while the other end was held by a monofilament snare around the muscle block. Both ends were connected to micromanipulators to allow for positioning of the trabecula and changing length. The super‐fusate was then switched to a 2,3‐butanedione 2‐monoxime‐free KH buffer containing 1 mM CaCl_2_ and 5 mM sodium pyruvate (see below), and field stimulation commenced (SD9, Grass Medical Instruments) using 5 ms pulses at a voltage 10% above threshold. Trabeculae were initially stimulated at 0.5 Hz, at room temperature. Once contracting regularly, trabeculae were lengthened until active isometric force was optimal (*L*_o_). Trabeculae diameter was then measured using a graticule and a 20 x objective, and cross‐sectional area (XSA) estimated assuming a cylindrical shape.

### 2.4 Composition of KH buffer solutions

KH buffer was prepared in 10 L batches of distilled water containing (mM): NaCl (118.0), KCl (4.75), MgSO_4_.7H_2_O (1.18), KH_2_PO_4_ (1.18), NaHCO_3_ (24.8). Immediately prior to experimentation, glucose (Allo et al., 1991) was added and the KH bubbled with carbogen (95% O_2_, 5% CO_2_) to bring pH to 7.4. For collection and transport of tissue, and during micro‐dissection of trabeculae, KH also contained (in mM): CaCl_2_ (0.25) and BDM (Power et al., 2020). During experimentation, sodium pyruvate (World Health Organization, 2016) and CaCl_2_ (1.5) were added to a BDM‐free KH super‐fusate.

### 2.5 Measurement of intracellular Ca^2+^

Intracellular calcium ([Ca^2+^]_i_) measurements were made as described previously (Power et al., 2018). Briefly, trabeculae were loaded for 2 h at room temperature (21.0 ± 0.1°C) in KH buffer with 10 μM fura‐2 am (Invitrogen, Thermofisher Scientific) and Powerload™; diluted 1:100 (Molecular Probes™). A measure of [Ca^2+^]_i_ was obtained from a window (approximately 120 μm × 120 μm) centered over the central portion of the trabeculae using a monochromator and spectrophotometric system (Cairn Research). Emitted fluorescence (~510 nm) was obtained at excitation wavelengths of 340 and 380 nm.

Post fura‐2 loading, the super‐fusate was replaced with KH buffer with 1.5 mM CaCl_2_, and 5 mM sodium pyruvate added, and the temperature raised to 37°C. 1 mM probenecid (Invitrogen, Thermofisher Scientific) was added to all super‐fusate solutions to prevent loss of fura‐2 from the cytosol at 37°C (Di Virgilio et al., 1988). Unless otherwise stated, trabeculae were then stimulated to contract at 1 Hz throughout experimentation.

**FIGURE 1:**
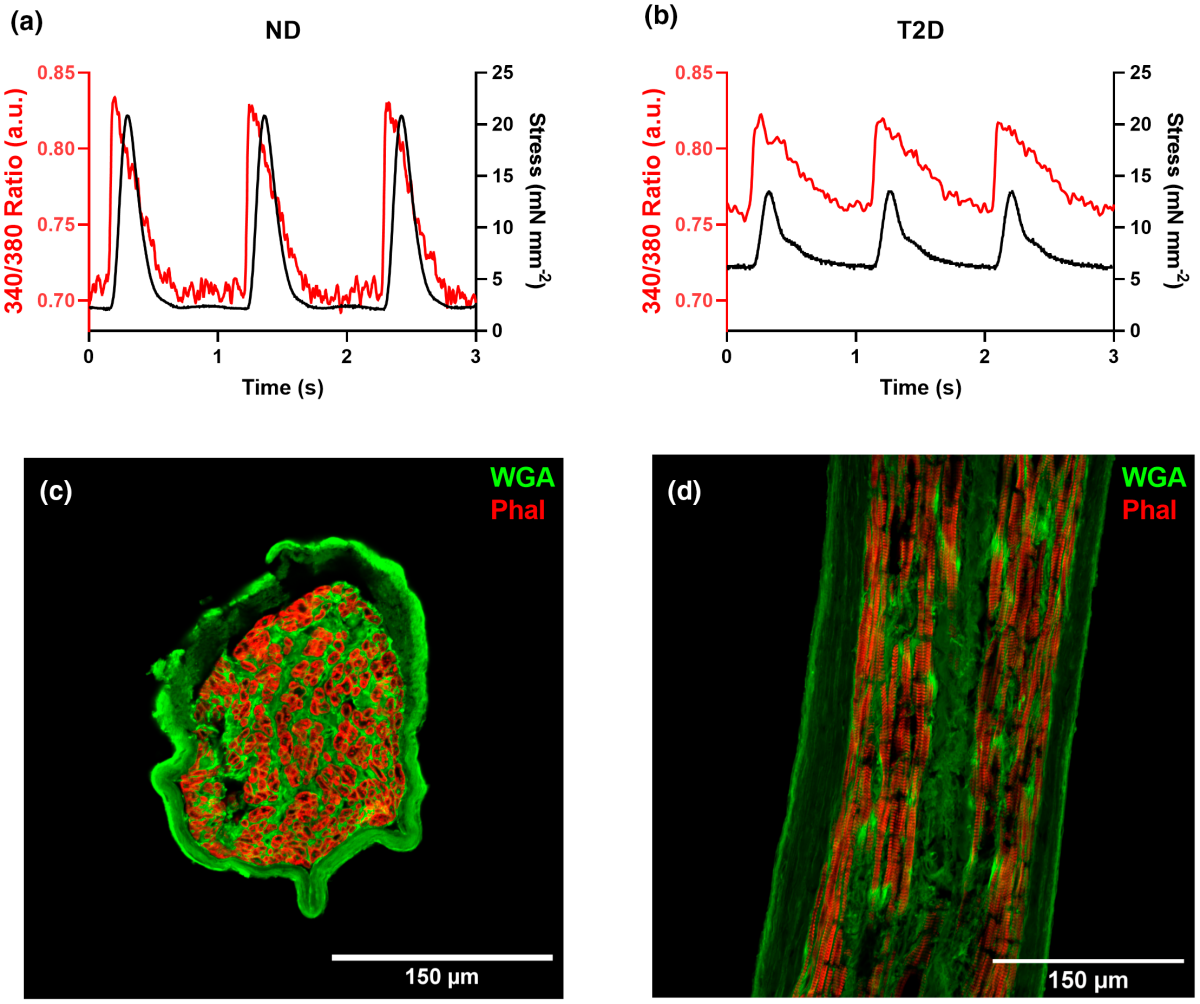
Cardiac trabeculae: structure and example data. (a) and (b) Representative trabeculae data from ND and T2D patients, respectively. Intracellular Ca^2+^ is shown in red, assessed by the 340/380 fura‐2 emitted fluorescence ratio. Contractile stress is shown in black, assessed by the force produced by the muscle, normalized to the total XSA of the muscle as determined by immunohistochemistry. (c, d) Example images of fixed trabeculae from non‐diabetic patients, labeled using immunohistochemistry in the transverse and longitudinal planes, respectively. Both sections were labeled with wheat germ agglutinin (WGA, green), labelling sialic acid residues on the cell membranes and extracellular matrix, and phalloidin (Phal, red), labelling cardiomyocyte myofilament F‐actin.

Ca^2+^ data are presented as either the 340/380 fura‐2 emitted fluorescence ratio (a. u.) or in nM determined using an in‐vivo calibration, as described previously (Ward et al., 2003). Isometric force was simultaneously obtained as a measure of contractile function and normalized to XSA to obtain stress (mN mm^−2^). Data was acquired using Acquisition Engine (Cairn Research). Representative data are shown in Figure 1a (ND) and 1B (T2D).

### 2.6 Data and statistical analysis

The stress and fluorescence data from 10 consecutive steady‐state cycles were exported as text files to be averaged and analyzed within a custom‐written IDL program (Research Systems Inc.). All data are expressed as mean ± SEM. Statistical significance was determined with Prism 8 (GraphPad Software) and either multivariate ANOVA with Holm‐Sidak multiple comparisons, Student's *t*‐test, or Fisher's exact test, used as appropriate. Statistical significance was determined as *p* ≤ 0.05. Trabeculae were divided into two groups based on their type 2 diabetic status.

### 2.7 Force‐frequency response and myofilament Ca^2+^ sensitivity

The physiological responsiveness of trabeculae was assessed in response to stimulation frequency, and myofilament Ca^2+^ sensitivity determined (Kaur et al., 2016; Power et al., 2018, 2020; Shen et al., 2013; Varian et al., 2006). Stimulation frequencies of 0.2, 0.5, 1, 1.5, 2, and 3 Hz were presented in a random order, and the trabeculae allowed to equilibrate to each new frequency before measurements of stress and intracellular Ca^2+^ were obtained.

The use of phase plots from intact cardiac trabeculae is an established method of determining myofilament Ca^2+^ sensitivity (Backx et al., 1995; Varian et al., 2006; Ward et al., 2003). The average of 10 consecutive Ca^2+^ transients and their associated contractions were obtained. Fura‐2340/380 ratio was calibrated to intracellular Ca^2+^ concentration (for description of method see Ward et al., 2003). Contractile stress and intracellular Ca^2+^ were then plotted against one another to produce a phase plot (shown in Figure 4a). Myofilament Ca^2+^ sensitivity was quantified by fitting a curve (Equation 1: Methods S1) to the relaxation component of the phase plots and the effective concentration (EC_50_) of intracellular Ca^2+^ at 50% of maximal stress obtained. Myofilament Ca^2+^ sensitivity was then compared between groups at a stimulation frequency of 1 Hz.

A second method was also utilized for determination of myofilament Ca^2+^ sensitivity, as previously described by Varian et al. (2006), King (2012) and Kaur et al. (2016), Mulieri et al. (1989). For this method trabeculae were super‐fused with a modified KH buffer containing (in mM): KCl (142), KH_2_PO_4_ (1.2), NaHCO_3_ (Power et al., 2020), MgSO_4_.7H_2_O (1.2), caffeine (Allo et al., 1991), glucose (Allo et al., 1991), CaCl_2_ (World Health Organization, 2016), in the absence of stimulation. The high K^+^ solution was applied for 1 minute before being replaced with standard (control) KH buffer. As the high K^+^ solution was washed out with control KH, trabeculae force and [Ca^2+^]_i_ both reduced toward baseline. The extended range of force and [Ca^2+^]_i_ data obtained from this protocol were then fitted with a modified Hill plot (Equation 2: Methods S1) to determine the EC_50_ (Figure 4e) (Kaur et al., 2016; Varian et al., 2006). As for the phase plots above, the EC_50_ of this curve was used to assess myofilament Ca^2+^ sensitivity and compare between groups.

### 2.8 Immunohistochemistry

On completion of functional studies, trabeculae were fixed and muscle structure examined using immunohistochemistry (Munro et al., 2018), in order to quantify the relative contributions of extracellular and intracellular changes due to type 2 diabetes. Trabeculae were held at optimal length and fixed in a solution containing 2% paraformaldehyde in phosphate‐buffered saline (PBS) for 10 min. Following fixation, trabeculae were sequentially dehydrated with 10%, 20% and 30% sucrose in PBS for 1 h per solution. Trabeculae were then placed in cryomolds, embedded in an optimal cutting temperature compound (Tissue Tek® Sakura Finetek Inc.), and frozen in 2‐methylbutane cooled with liquid nitrogen. Trabeculae were stored at −80°C until cryostat sectioning (CM3050, Leica) at a thickness of 10 μm. Care was taken to ensure transverse sections were obtained from the central portion of the trabeculae. Sections were collected on poly‐L‐lysine coated coverslips to ensure adhesion, in preparation for antibody labelling.

#### 2.8.1 Myofilament cross‐sectional area labelling

Trabeculae sections were rehydrated in PBS for 5 min before blocking for 1 hour with Image iT FX Signal Enhancer (Thermofisher Scientific). Pre‐conjugated Alexa‐488 wheat‐germ agglutinin (WGA, 1:200, Thermofisher Scientific) and Alexa‐594 phalloidin (1:50) was diluted in 1% bovine serum albumin (BSA), 0.01% NaN_3_, and 0.05% Triton‐X100 in PBS and applied to sections for 2 h at room temperature. The coverslips were then mounted on slides using Prolong Gold (Thermofisher Scientific) and left to cure for 72 h at 4°C.

Antibody‐labeled sections were imaged using a Zeiss 710 laser scanning confocal microscope (Zeiss) using ZEN software (Zeiss). Images taken for myofilament XSA measurements were acquired using either a Zeiss 10× (NA: 0.45) or 20× (NA: 0.8) air objective (depending on whether the entire section could be captured in the field of view). Images were analyzed using Fiji (Image J). Trabeculae XSA was determined by tracing the perimeter of WGA labelling within each myocyte. Myofilament XSA was obtained by summing the total area of phalloidin labelling per trabecula, also calculated using Fiji (Figure 3d). Three trabeculae from the ND group did not yield suitable sections for analysis of XSA by confocal microscopy, so estimation of their XSA was based on the light microscopy measurement of muscle diameter during experimentation.

## 3 RESULTS

### 3.1 Patient clinical data

Viable trabeculae were obtained from *n* = 11 ND patients and *n* = 12 T2D patients. Table 1 provides a summary of patient data for each group. There were no significant differences in any of the measured clinical characteristics between groups (with the exception of HbA_1c_). Diabetic status of patients was obtained from their clinical notes. Patients with an HbA1c of 40 mmol/mol or higher were assigned to the T2D group. HbA1c was not recorded in the clinical notes of non‐diabetic patients.

**TABLE 1:** Patient characteristics and medications.

	ND (*n* = 11)	T2D (*n* = 12)
Age (years)	68.8 ± 2.7	61.0 ± 3.2
Gender (male/female)	3 Female 8 Male	2 Female 10 Male
BMI (kg/m^2^)	28.19 ± 1.47	29.17 ± 1.15
HbA1c (mmol/mol)	N/A	65.0 ± 6.6
Ejection fraction (%)	53.82 ± 2.40	49.50 ± 3.56
Cigarette smoker	5/11	8/12
Hypertension	7/11	11/12
Hyperlipidemia	9/11	11/12
ACE Inhibitors	6/11	7/12
AT1 Blockers	4/11	2/12
β‐blockers	5/11	9/12
Ca^2+^ channel blockers	3/11	4/12
Nitrates	5/11	8/12
Lipid lowering drugs	9/11	11/12
Diuretics	0/11	1/12
Insulin	0/11	0/12

*Note*: Data shown are mean ± SEM or as a proportion of sample size. There were no significant differences in age, BMI, or ejection fraction between groups. Student's *t*‐test was used to test for differences between groups.

### 3.2 Force‐frequency response data

**FIGURE 2:**
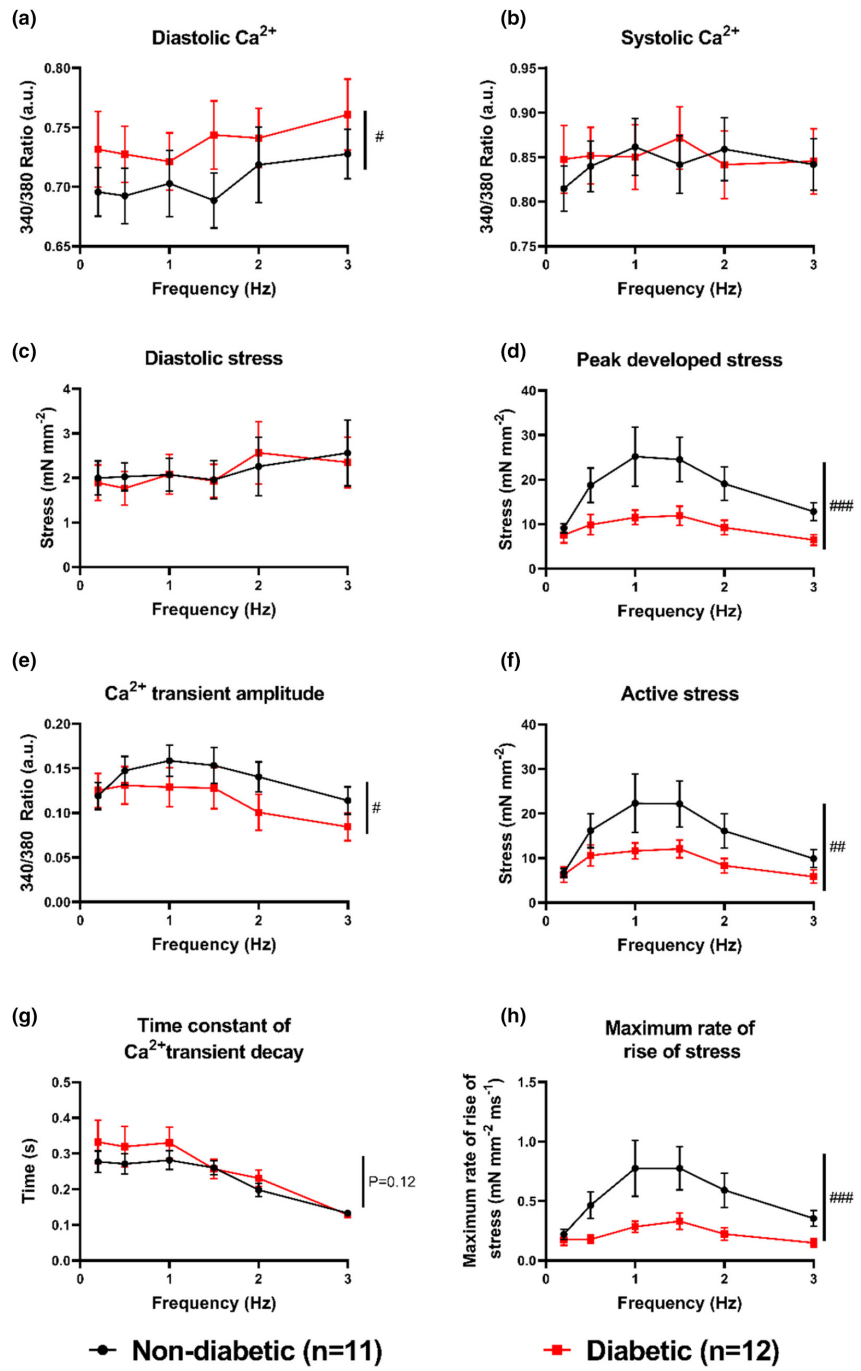
Trabeculae Ca^2+^ handling and contractile parameters during force–frequency response. Ca^2+^ handling and contractile parameters were obtained from each trabecula by averaging 10 consecutive Ca^2+^ transients, and their associated twitches, at six different stimulation frequencies. Mean ± SEM trabeculae data are shown from *n* = 11 ND and *n* = 12 T2D patients. Significance was determined by two‐way ANOVA, with #*p* ≤ 0.05, and ###*p* ≤ 0.001 for the group effect. Ca^2+^ transient parameters are shown in panels: (a) diastolic, (b) systolic, (e) amplitude (peak minus diastolic), and the (g) time constant of Ca2+ decay. Contractile parameters are shown in panels: (c) diastolic stress, d) peak developed stress, (f) active (systolic minus diatolic) stress, and (h) the maximum rate of rise in stress.

Figure 2 shows mean ± SEM steady‐state data from trabeculae stimulated at 0.2, 0.5, 1, 1.5, 2, and 3 Hz. Trabeculae from T2D patients had significantly higher diastolic (resting) Ca^2+^ (*p* = 0.03, Figure 2a), no difference in peak systolic Ca^2+^ (*p* = 0.54, Figure 2b), and significantly smaller Ca^2+^ transient amplitudes (*p* = 0.04, Figure 2e). No significant difference was observed in the time to peak Ca^2+^ (*p* = 0.27, Table S3) or the time constant of Ca^2+^ transient decay *p* = 0.12, Figure 2g). The maximum rate of rise of fluorescence was significantly lower in trabeculae from T2D patients (*p* ≤ 0.001, Table S3). There were no significant differences in the time to peak stress (*p* = 0.30, Table S3), the time to 50% twitch relaxation (*p* = 0.50, Table S3) or the time to 90% twitch relaxation (*p* = 0.14, Table S3), however the maximum rate of rise of stress was significantly lower in trabeculae from T2D patients (*p* ≤ 0.0001, Figure 2h). Trabeculae from T2D patients did not show any significant differences in diastolic stress (normalized to total trabecula XSA, (*p* = 0.87, Figure 2c) but did display significantly reduced peak developed stress (normalized to total trabecula XSA, *p* ≤ 0.0001, Figure 2d) and active stress (normalized to total trabecula XSA, *p* ≤ 0.001, Figure 2f).

### 3.3 Immunohistochemistry

**FIGURE 3:**
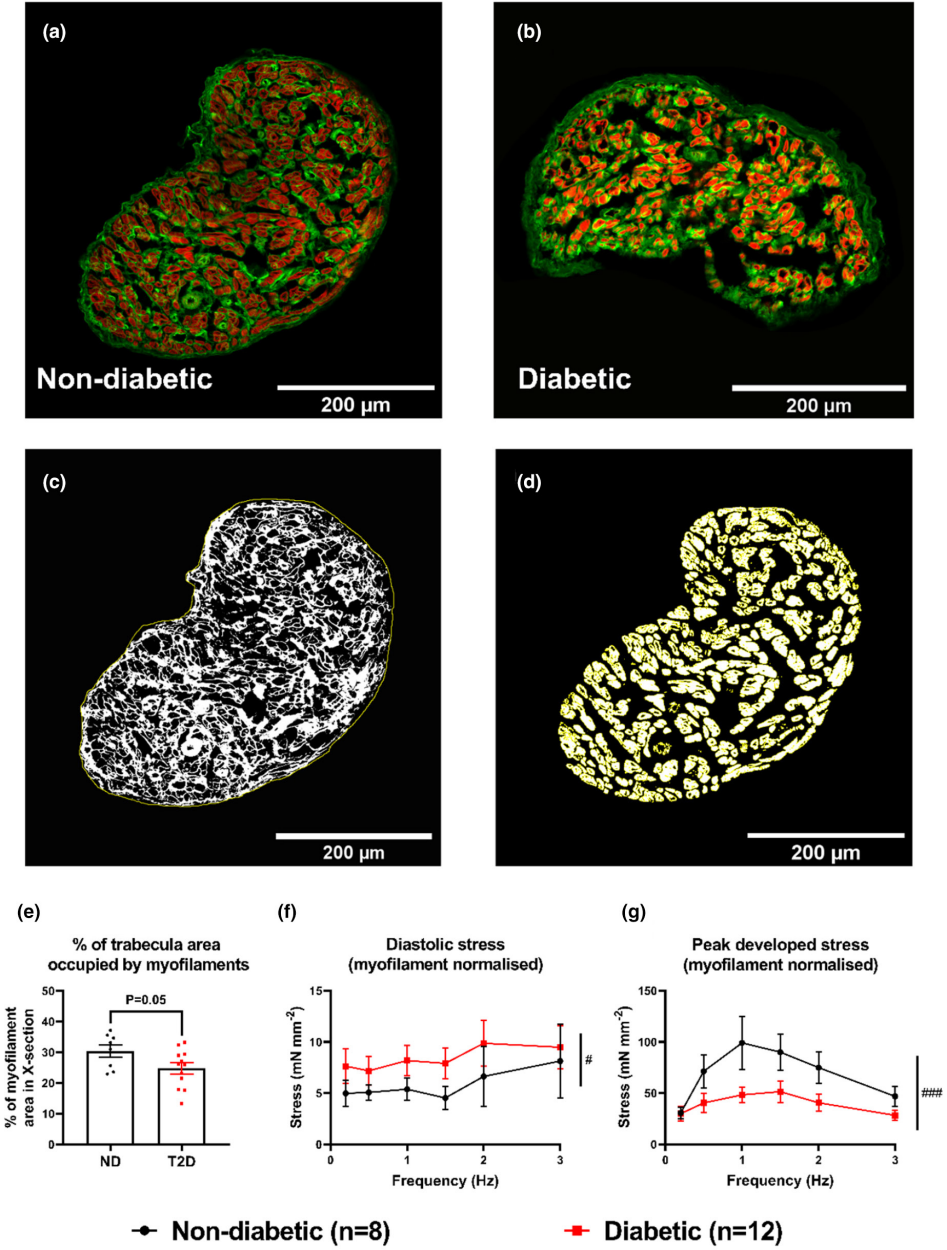
Immunohistochemical analysis of trabeculae cross‐sectional area. 10 μm thick sections of human RAA trabeculae imaged on a Zeiss LSM 710 confocal microscope. (a) and (b) Examples of images of trabeculae from ND and T2D patients, respectively. Sialic acid residues labeled with WGA are shown in green while myofilament f‐actin labeled with phalloidin is shown in red. (c) The method of measuring total trabecula XSA by tracing the outline of the WGA labelling (shown in white). (d) The method of measuring myofilament XSA by outlining the phalloidin labelling and measuring the area within using Fiji. (e) The percentage of total XSA occupied by myofilaments from eight trabeculae from ND patients and 11 trabeculae from T2D patients. (f) and (g) The diastolic and peak developed stress, respectively during the force frequency response normalized to myofilament XSA. All data are presented as mean ± SEM with significance determined by unpaired t‐tests, or two‐way ANOVA, with #representing *p* ≤ 0.05 and ###*p* ≤ 0.001 for the group effect.

The light microscope was used to determine trabeculae XSA during experimentation by measuring the width at L_o_ from above, and assuming they were cylindrical in shape. More accurate measurements of trabeculae XSA, and the myofilament area per trabeculae cross‐section were subsequently obtained using confocal microscopy of fixed trabeculae cross sections labeled with phalloidin. The total XSA (ND: 0.050 ± 0.009 mm^2^; T2D: 0.081 ± 0.014 mm^2^, *p* = 0.12) and the myofilament XSA (ND: 0.016 ± 0.003 mm^2^; T2D: 0.019 ± 0.003 mm^2^, *p* = 0.51) were not significantly different between groups. However, T2D trabeculae showed a lower percentage of total XSA occupied by myofilaments (ND: 30.5 ± 2.0%; T2D: 24.8 ± 1.8%, *p* = 0.05, Figure 3c). No significant difference was found between the groups for XSA obtained with either method (ND: Light microscope estimate = 0.062 ± 0.010 mm^2^; Immunohistochemistry measurement = 0.050 ± 0.009 mm^2^, *p* = 0.41. T2D: Light estimate = 0.058 ± 0.007 mm^2^; Immunohistochemistry measurement = 0.081 ± 0.014 mm^2^, *p* = 0.07). When trabeculae were not divided into groups based on diabetic status, there were still no significant differences between the methods (Light estimate = 0.060 ± 0.006 mm^2^; Immunohistochemistry measurement = 0.069 ± 0.010 mm^2^, *p* = 0.39).

Normalization of contractile force to *myofilament* XSA allows for isolation of cardiomyocyte performance rather than as a component of cardiac tissue with varying amounts of extracellular matrix. At 1 Hz, trabeculae from T2D patients showed a significantly lower maximum rate of rise of stress normalized to myofilament XSA: ND: 3.131 ± 0.843 mN mm^−2^ ms^−1^; T2D: 1.360 ± 0.219 mN mm^−2^ ms^−1^, *p* = 0.03, (Table S2), and significantly lower active stress when normalized to myofilament XSA: ND: 96.52 ± 26.36 mN mm^−2^; T2D: 44.20 ± 7.47 mN mm^−2^, P = 0.04, (Table S2). Three trabeculae from ND patients were excluded from this analysis due to a lack of usable transverse sections for obtaining myofilament XSA by confocal microscopy.

Normalization of stress to *myofilament* XSA, instead of total XSA, during the force‐frequency response measurements was also examined. Trabeculae from T2D patients had significantly higher diastolic stress (*p* ≤ 0.008, Figure 3d), significantly lower peak developed stress (*p* ≤ 0.001, Figure 3e) and active stress (*p* ≤ 0.001, data not shown).

### 3.4 Myofilament Ca^2+^ sensitivity

**FIGURE 4:**
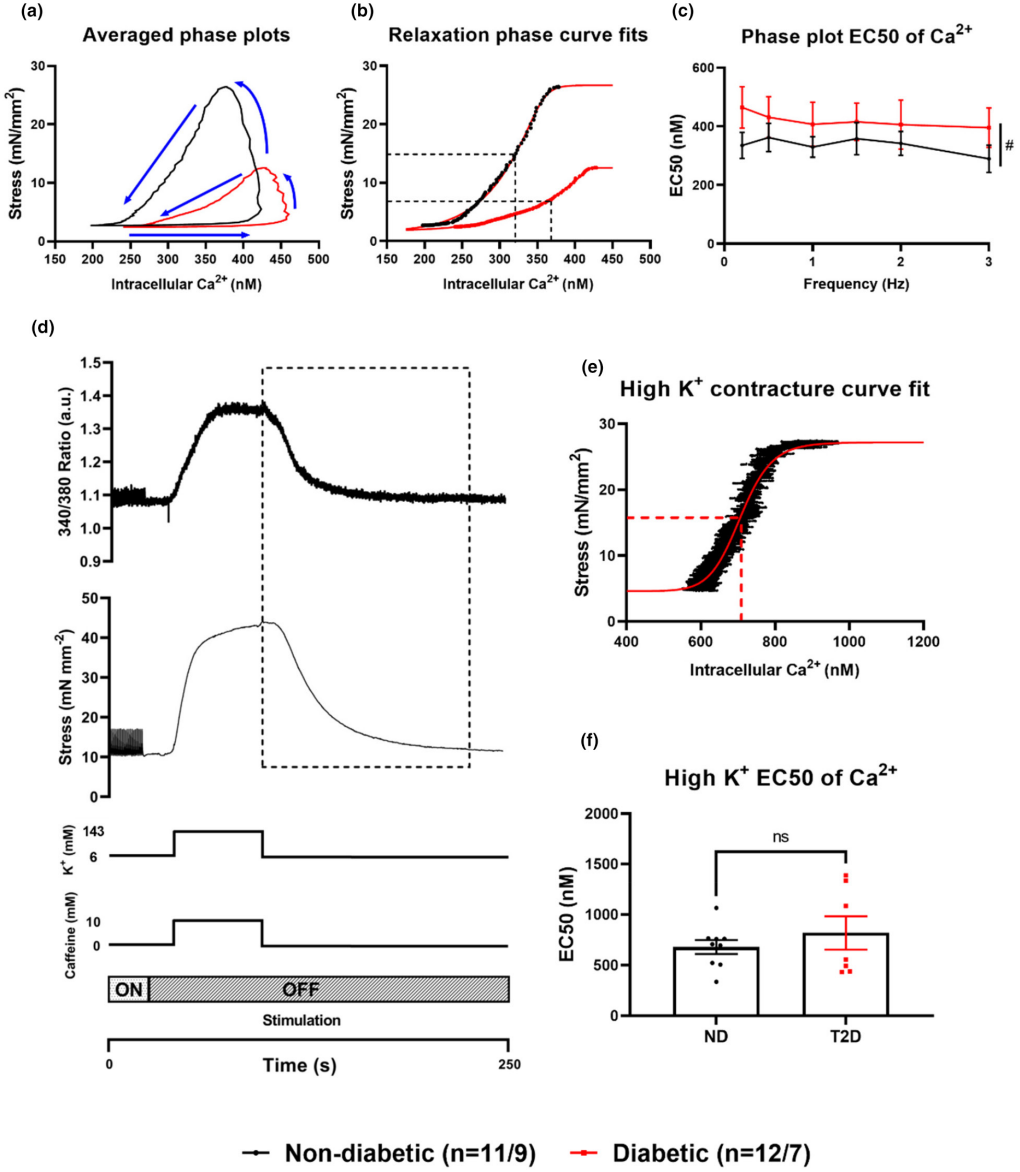
Myofilament Ca^2+^ sensitivity. Assessment of myofilament Ca^2+^ sensitivity through either phase plots generated from Ca^2+^ transients and associated contractions during the force‐frequency response, or from exposure to high [K^+^] contractures. (a) Representative phase plots from averaged data at 1 Hz in trabeculae from patients with (red), and without (black) diabetes. (b) The curve fits for the relaxation component of the phase plots in (a), with dotted lines for the calculated EC_50_ values. (c) The calculated EC_50_ values from the curve fits during the force frequency response in trabeculae from ND (*n* = 11) and T2D (*n* = 12) patients. (d) An example of a high K^+^ contracture with the dotted rectangle representing the relaxation phase used for curve fitting. (e) An example of curve fitting for a representative high K^+^ contracture. (f) The calculated EC_50_ mean values of the high K^+^ contractures in trabeculae from T2D (*n* = 7) and ND (*n* = 9). Data are presented as mean ± SEM with significance determined by two‐way ANOVA, with # representing *p* ≤ 0.05 for the group effect.

The change in myofilament Ca^2+^ sensitivity was tested using either phase plots of force against [Ca^2+^]_i_ for a range of stimulation frequencies, or by exposing the trabeculae to super‐fusion with a high [K^+^] and monitoring the recovery following return to control KH with [K^+^] of 6 mM. There was no difference in the EC_50_ of the fitted curves applied to the relaxation portion of the phase plots due to stimulation frequency in either group (*p* = 0.94, Figure 4c), however trabeculae from patients with T2D (*n* = 12) had significantly higher EC_50_ values compared to trabeculae from ND (*n* = 11) patients (*p* = 0.018, Figure 4c) indicating a reduction in myofilament Ca^2+^ sensitivity. There was no significant difference in the EC_50_ of the fitted curves applied to the relaxation phase of the high K^+^ contractures between trabeculae from ND patients (*n* = 9) and trabeculae from T2D patients (*n* = 7) (ND: 679.7 ± 69.0 nM; T2D: 818.6 ± 163.9 nM, *p* = 0.41, Figure 4f).

## 4 DISCUSSION

### 4.1 Patient clinical characteristics

Research using isolated cardiac tissue from humans frequently lacks access to healthy “control” tissue for comparison, particularly when samples are obtained from patients in a hospital environment. Patients recruited for this study were all undergoing coronary artery bypass grafting surgery. However, the ND and T2D study groups had no significant difference in any of the clinical parameters measured (Table 1) with the exception of HbA_1c_ which was used to determine diabetic status. While it is difficult to know whether any of the medications that patients were taking prior to surgery would have had an effect on the isolated trabeculae data obtained, there were no significant differences between the groups in the proportions of patients taking any particular class of medication. We expect there to be no residual effect of any patient medications on the trabeculae measurements made since continuous super‐fusion with K‐H buffer would rapidly remove any remaining drug from such tiny muscle preparations (XSA <0.1 mm^2^).

### 4.2 Structural differences in trabeculae

Unlike trabeculae studies utilizing ventricular trabeculae from young adult rat hearts (Power et al., 2018; ter Keurs et al., 1980; Ward et al., 2003), human atrial trabeculae showed significant variability in the composition of the muscles, with some possessing an abundance of cardiomyocytes (Figure 3a) and others having relatively few (Figure 3b). We therefore normalized our stress data to contractile protein content for comparisons between groups. Human atrial trabeculae also frequently showed an increased layer of connective tissue encasing the trabeculae, not commonly observed in rat ventricular trabeculae, perhaps a result of the patient cohort age. Nevertheless, our study shows the functional performance of human atrial trabeculae was comparable to that produced by rat ventricular trabeculae (Power et al., 2018, 2020; Ward et al., 2003). This difference in the composition of the muscles highlights the importance of structural analysis and that simple normalization of force to total muscle XSA is insufficient if the goal of the experiments is to assess the contractile ability of the *cardiomyocytes* themselves. Differences in the percentage of muscle XSA occupied by non‐myocytes between ND and T2D patients (Figure 3e) may be due to increased cardiac fibrosis in the atria of patients with diabetes (Regan et al., 1977; Russo & Frangogiannis, 2016; van Heerebeek et al., 2008). While differences in trabeculae composition would contribute to the observed contractile deficits in trabeculae from T2D patients, the normalization of contractile force to myofilament area of trabeculae (Figure 3d) did not eliminate the contractile differences between ND and T2D groups (Figure 3f,g). This confirms that *cardiomyocyte* function is reduced in the atria of diabetic patients, rather than reduced contractility in diabetes being limited to the impact of fibrosis.

### 4.3 Calcium handling and contractile stress in trabeculae from diabetic patients

Our immunohistochemical results showed that differences in trabeculae structure could not account for the contractile deficits observed in trabeculae from T2D patients, therefore there must be *intracellular* deficits within the cardiomyocytes. Trabeculae from T2D patients had increased diastolic Ca^2+^ (Figure 2a), without differences in peak systolic Ca^2+^ (Figure 2b), resulting in an overall decrease in the amplitude of the Ca^2+^ transients (Figure 2e). Given the importance of Ca^2+^ in determining crossbridge cycling and force development, the reduced Ca^2+^ transient amplitude can explain the observed decrease in peak developed (Figure 2d) and active stress (Figure 2f) in trabeculae from T2D patients. While the diastolic stress (Figure 2c) obtained from force normalized to total trabeculae XSA was not different between group, when normalized to *myofilament* XSA it was significantly increased in trabeculae from T2D patients (Figure 3f).

Figure 3e shows trabeculae from diabetic patients had reduced myofilament content as a percentage of XSA. If the non‐myofilament portion of diabetic trabeculae was due to *fibrosis*, then we would expect them to show *increased* diastolic stress in comparison to non‐diabetic trabeculae. Particularly since diabetic trabeculae also had increased diastolic [Ca^2+^]_i_ (Figure 2a). However, we found diastolic stress was not different between groups when normalized to trabeculae XSA (Figure 2c), and was *increased* (Figure 3f) in diabetic trabeculae when normalized to myofilament area alone. These data suggest that the diastolic [Ca^2+^] (Figure 2a) in trabeculae from diabetic patients had a much greater contribution to diastolic stress than *either* the non‐myocyte area (which may not have been due only to fibrosis) *or* the reduced myofilament Ca^2+^ sensitivity. Trabeculae from T2D patients also displayed a slower maximum rate of rise of fluorescence (Table S3), which was matched by a slower maximum rate of rise of stress in trabeculae from T2D patients (Figure 2h), which may suggest defective calcium current and/or excitation‐contraction coupling.

While the changes to Ca^2+^ handling were significant, they were not as dramatic as the changes in contractility, with a ~50% decrease in peak developed stress at certain stimulation frequencies (Figure 2d). It is unlikely that the reduction in Ca^2+^ transient amplitude could account for such a large decrease in contractility, implicating other components of excitation–contraction coupling may be at fault. Reduced myofilament Ca^2+^ sensitivity was also observed in trabeculae from T2D patients using two different experimental approaches. High [K^+^] added to the KH buffer trigger trans‐sarcolemmal influx of Ca^2+^ into the cytosol which is then combined with caffeine‐induced release of Ca^2+^ from the SR to achieve much higher concentrations of cytosolic Ca^2+^ than under physiological conditions. Although this intervention yielded a similar decrease in myofilament Ca^2+^ sensitivity to the analysis of phase plot relaxation, it was not statistically significant. However, myofilament sensitivity determined from phase plots obtained over a range of stimulation frequencies showed a significantly reduced sensitivity in trabeculae from diabetic patients (Figure 4c), as previously reported in diabetic heart disease (Akella et al., 1995; Hofmann et al., 1995; Ward & Crossman, 2014; Zhang et al., 2008). Decreased myofilament Ca^2+^ sensitivity in rodent models of diabetes has been attributed to multiple factors including O‐linked N‐acetylglucosamine(O‐GlcNAc)‐ylation of serine and threonine residues on actin, troponin‐I, myosin heavy chain, and myosin light chain (Ramirez‐Correa et al., 2008, 2015), supported by the observation of significant upregulation of O‐GlcNAcylation of protein in diabetes (Erickson et al., 2013; Marsh et al., 2014).

## 5 CONCLUSIONS

In conclusion, this study has simultaneously measured changes in intracellular Ca^2+^ and contractile stress in human cardiac tissue. This study displays that the contractile dysfunction observed in diabetic heart disease is multifactorial but cannot be explained solely by changes of the extracellular matrix. While there were significant changes to the composition of the RAA trabeculae in patients with type 2 diabetes, the contractile dysfunction likely included contribution from disruption of cardiomyocyte Ca^2+^ handling and reduction of myofilament Ca^2+^ sensitivity. This study identifies the need to assess the way that cardiomyocytes handle Ca^2+^ in diabetic heart in more detail as well as the importance of examining the cellular composition of isolated muscles when using multicellular preparations. The identification of the disruption of Ca^2+^ handling may open the way for more precise treatment strategies, targeting the cellular mechanisms of contractile impairment in diabetic patients, in order to improve patient quality of life and reduce mortality.

## AUTHOR CONTRIBUTIONS

M‐LW conceived and supervised the project. TLMJ conducted the experiments, analyzed the data and acquired the tissue samples. SK provided experimental assistance and advice. PNR and NK provided clinical insight, and NK assisted in tissue acquisition. M‐LW and TLMJ wrote the manuscript. All authors commented on and contributed to the final manuscript.

## FUNDING INFORMATION

This work was supported by the University of Auckland [FMHS R&D fund to M‐LW]; and the Auckland Medical Research Foundation [1118006 to M‐LW, 1121010 to M‐LW]; and the Maurice and Phyllis Paykel Trust [Research project to M‐LW]; and the Greenlane Research and Education Fund [18/16/4139 to PNR], and the National Heart Foundation of New Zealand [doctoral scholarship 1737 to TLMJ].

## CONFLICT OF INTEREST STATEMENT

The authors declare no conflicts of interest.

## Supporting information


Data S1
