# Supplementary material for: Impaired calcium handling mechanisms in atrial trabeculae of diabetic patients
Source: Physiol Rep. 2023 Feb 7;11(3):e15599. doi: 10.14814/phy2.15599 (PMC9904963; doi:10.14814/phy2.15599)
Supplement: Supplementary file 1 — Data S1 [file PHY2-11-e15599-s001.docx]

Supplementary methods and material

Chemicals and solutions

Chemicals were all sourced from Sigma-Aldrich (USA), unless otherwise specified.

Human right atrial appendage tissue samples

Patients awaiting routine coronary artery bypass grafting surgery at Auckland Hospital were recruited to the study and provided informed consent for removal of a small section (approximately 0.5 cm by 2 cm) of right atrial appendage (RAA) tissue. Tissue was excised from the border of the cannula incision site prior to atrial cannulation, and immediately transferred to 100 mL of Krebs-Hensleit (KH) buffer oxygenated with 95% O_2_, 5% CO_2_. The sample was then transferred to our laboratory at the University of Auckland. The mean ± SEM time between removal of tissue from the patient to commencing superfusion with fresh buffer in the laboratory was 7 ± 1 minutes.

Patients were retrospectively classified as diabetic (T2D) if their glycated haemoglobin (HbA1c) was 40 mmol/mol or higher, and non-diabetic (ND) if their HbA1c was < 40 mmol/mol.

Microdissection of trabeculae

Tissue samples were pinned out in a dissection dish and kept continuously oxygenated with fresh buffer solution. When present, an unbranched trabecula 2-4 mm long and less than 300 µm in diameter was micro-dissected from the endocardial surface of RAA tissue and mounted in a temperature controlled muscle chamber (Aurora Scientific, Canada), on the stage of an inverted microscope (Nikon Eclipse TE2000-U, Tokyo, Japan). The tissue block at one end of the trabecula was held in a wire basket extending from the beam of a force transducer (AE801, Kronex Technologies, USA) while the muscle block at the other end was held by a monofilament snare. Both ends were connected to micromanipulators to allow for positioning of the trabecula and changing length. Trabeculae were continuously superfusesd with oxygenated KH buffer solutions throughout experimentation. The superfusate was then switched to a 2,3-butanedione 2-monoxime -free KH buffer containing 1 mM CaCl_2_ and 5 mM sodium pyruvate, and field stimulation commenced (SD9, Grass Medical Instruments, Quincy, MA, USA) using 5ms pulses at a voltage 10% above threshold. Trabeculae were initially at room temperature, and stimulated at 0.5 Hz. Once contracting regularly, trabeculae were lengthened until active isometric force was optimal (L_0_). Trabeculae diameter was then measured using a graticule and the 20 x objective, and XSA estimated assuming a cylindrical shape.

Measurement of intracellular Ca^2+^

Intracellular calcium ([Ca^2+^]_i_) measurements were made as described previously [21]. Briefly, trabeculae were loaded for 2 hours at room temperature (21.0 ± 0.1 °C) with 10 µM fura-2 AM (Invitrogen, Thermofisher Scientific, USA) and Powerload^TM^; diluted 1:100 (Molecular Probes^TM^, Oregon, USA). A window (approximately 120 µm by 120 µm) centred over the central portion of the trabeculae was used for [Ca^2+^]_i_ measurements using a monochromator and spectrophotometric system (Cairn Research, Faversham, UK). Emitted fluorescence (~510 nm) was obtained at excitation wavelengths of 340 nm and 380 nm.

Post fura-2 loading, the superfusate was replaced with KH buffer containing 1.5 mM CaCl_2_, and 5 mM sodium pyruvate. The temperature was raised to 37°C and 1 mM probenecid (Invitrogen, Thermofisher Scientific, USA) was added to all superfusate solutions to prevent loss of fura-2 from the cytosol at 37°C (16). Unless otherwise stated, trabeculae were then stimulated to contract at 1 Hz throughout experimentation.

Ca^2+^ data is presented as either the 340/380 fura-2 emitted fluorescence ratio, or in nM determined using an in-vivo calibration, as described previously (17). Isometric force was simultaneously obtained as a measure of contractile function and normalised to trabeculae cross-sectional area (XSA) to obtain stress (mN mm^-2^).

Data and statistical analysis

Data were acquired using Acquisition Engine (Cairn Research, Faversham, UK). The stress and fluorescence data from 10 consecutive steady-state cycles were exported as text files to be averaged and analysed within a custom-written IDL program (Research Systems Inc., Boulder, CO, USA). All data are expressed as mean ± SEM. Statistical significance was determined using Prism 8 (GraphPad Software, USA) and either multivariate ANOVA with Holm-Sidak multiple comparisons, Student’s t-test, or Fisher’s exact test, as appropriate, Statistical significance was determined as *p* ≤ 0.05. Trabeculae were divided into two groups based on their type 2 diabetic status.

Force-frequency, and myofilament Ca^2+^ sensitivity

The response of trabeculae to stimulation frequencies of 0.2, 0.5, 1, 1.5, 2 and 3 Hz was investigated. Stimulation frequencies were presented in a random order, and the trabeculae allowed to equilibrate to the new frequency before measurements of stress and intracellular Ca^2+^ concentration were obtained.

Myofilament Ca^2+^ sensitivity was quantified by measuring the half maximal effective concentration (EC_50_) of intracellular Ca^2+^. This parameter is equal to the Ca^2+^ concentration when contractile stress is halfway to maximum from its baseline during the relaxation phase of the phase plots. This EC_50_ was obtained by fitting using an asymmetrical 5-parameter dose response curve to the relaxation phase of the phase plots. This curve is described by the equation below:

$F = F_{min}+ \frac{F_{max}-F_{min}}{\left( 1+\left( 2^{\frac{1}{S}}-1 \right)\times\left( \frac{EC50}{\left[ {Ca}^{2+} \right]} \right)^{h} \right)^{S}}$ [Equation 1]

Where F equals the contractile stress,

F_max_ equals the maximum contractile stress,

F_min_ equals the minimum contractile stress,

[Ca^2+^] equals the intracellular Ca^2+^ concentration,

EC_50_ equals the intracellular Ca^2+^ concentration at 50% of the active stress $\left( \frac{\left( F_{max}-F_{min} \right)}{2}+F_{min} \right)$,

*h* equals a unit-less Hill slope parameter used to determine the slope of the curve,

*S* equals a unit-less symmetry parameter used to determine the symmetry of the curve.

The curves were fitted using GraphPad Prism 9 which fitted the curves by determining the *h* and *S* parameters where the R-squared value of the fits was highest. The EC_50_ of these curves was used as the measurement of myofilament Ca^2+^ sensitivity.

Myofilament Ca^2+^ sensitivity was also quantified using a method previously described by Varian et al. 2006 and Kaur et al. 2016. A modified KH buffer containing (in mM): KCl (142), KH_2_PO_4_ (1.2), NaHCO_3_ (20), MgSO_4_.7H_2_O (1.2), caffeine (10), glucose (10), CaCl_2_ (5) was applied to trabeculae in the absence of stimulation. The high K^+^ concentration of the buffer triggers a very large trans-sarcolemmal influx of Ca^2+^ into the cytosol, combined with release of Ca^2+^ from the SR caused by caffeine application. The high K^+^ solution was applied for 1 minute before being replaced with a normal KH buffer. The high Ca^2+^ concentration resulted in an abnormally large contraction, thus providing a second method for the assessment of myofilament Ca^2+^ sensitivity between groups. As the high K^+^ solution was washed out with control KH, trabeculae relaxed towards baseline. The relaxation phase of the contracture was then fitted with a modified Hill plot to determine the EC_50_ (Varian et al., 2006; Kaur et al., 2016). As above the EC_50_ of this curve was used to assess myofilament Ca^2+^ sensitivity and compare between groups. The equation for this curve is described below:

$F = F_{min}+ F_{max}\left( \frac{\left[ {Ca}^{2+} \right]^{h}}{{EC50}^{h}+\left[ {Ca}^{2+} \right]^{h}} \right)$ [Equation 2]

Where:

F equals the contractile stress,

F_max_ equals the maximum contractile stress,

F_min_ equals the minimum contractile stress,

[Ca^2+^] equals the intracellular Ca^2+^ concentration,

EC_50_ equals the intracellular Ca^2+^ concentration at 50% of the active stress $\left( \frac{\left( F_{max}-F_{min} \right)}{2}+F_{min} \right)$,

*h* equals a unit-less Hill slope parameter used to determine the slope of the curve.

As above the EC_50_ of this curve was used to assess myofilament Ca^2+^ sensitivity and compare between groups.

|  | Non-diabetic (n=8) | Diabetic (n=12) | P value |
| --- | --- | --- | --- |
| Trabeculae XSA area (mm^2^) | 0.050 ± 0.009 | 0.081 ± 0.014 | 0.12 |
| Myofilament/trabeculae XSA (mm^2^) | 0.016 ± 0.03 | 0.019 ± 0.003 | 0.51 |
| Maximum rate of rise of stress (mN mm^-2^ ms^-1^) | 0.784 ± 0.218 | 0.325 ± 0.049 | **^#^ 0.049** |
| Maximum rate of rise of stress normalised to myofilament XSA (mN mm^-2^ ms^-1^) | 3.131 ± 0.843 | 1.360 ± 0.219 | **^#^ 0.03** |
| Diastolic stress  (mN mm^-2^) | 2.65 ± 0.49 | 2.35 ± 0.71 | 0.74 |
| Diastolic stress normalised to myofilament XSA (mN mm^-2^) | 6.38 ± 1.33 | 9.26 ± 2.61 | 0.35 |
| Peak developed stress (mN mm^-2^) | 27.27 ± 7.11 | 12.94 ± 2.00 | 0.06 |
| Peak developed stress normalised to myofilament XSA (mN mm^-2^) | 103.79 ± 27.96 | 53.45 ± 8.69 | 0.06 |
| Active stress (mN mm^-2^) | 24.62 ± 6.91 | 10.58 ± 1.67 | **^#^ 0.05** |
| Active stress normalised to myofilament XSA (mN mm^-2^) | 96.52 ± 26.36 | 44.20 ± 7.47 | **^#^ 0.04** |

**Table 2. Normalisation of trabeculae contractile parameters at 1Hz steady state to myofilament cross-sectional area.**

Contractile parameters obtained from averaging 10 Ca^2+^ transients and their associated twitches from n = 8 trabeculae from patients without diabetes and n= 12 from patients with diabetes, with and without normalisation to myofilament XSA. Data presented as mean ± SEM. Significance was determined by Students t-test. **^#^** = P ≤ 0.05

|  | **ND (n = 11)** | | | | | | **T2D (n = 12)** | | | | | | **Two-way ANOVA Group Effect (P-value)** |
| --- | --- | --- | --- | --- | --- | --- | --- | --- | --- | --- | --- | --- | --- |
| **Frequency (Hz)** | **0.2** | **0.5** | **1** | **1.5** | **2** | **3** | **0.2** | **0.5** | **1** | **1.5** | **2** | **3** |  |
| **Time to peak Ca^2+^ (s)** | 0.060 ± 0.005 | 0.085 ± 0.015 | 0.067 ± 0.006 | 0.067 ± 0.006 | 0.066 ± 0.004 | 0.054 ± 0.003 | 0.054 ± 0.005 | 0.069 ± 0.006 | 0.091 ± 0.015 | 0.075 ± 0.011 | 0.088 ± 0.085 | 0.067 ± 0.007 | 0.27 |
| **Time constant of Ca^2+^ transient decay (s)** | 0.277 ± 0.030 | 0.271 ± 0.029 | 0.282 ± 0.026 | 0.260 ± 0.020 | 0.198 ± 0.019 | 0.131 ± 0.010 | 0.332 ± 0.061 | 0.320 ± 0.057 | 0.330 ± 0.044 | 0.257 ± 0.028 | 0.231 ± 0.023 | 0.129 ± 0.006 | 0.12 |
| **Maximum rate of rise of fluorescence (a.u. ms^-1^)** | 0.015 ± 0.002 | 0.016 ± 0.002 | 0.019 ± 0.002 | 0.021 ± 0.003 | 0.019 ± 0.002 | 0.018 ± 0.003 | 0.014 ± 0.003 | 0.013 ± 0.003 | 0.014 ± 0.003 | 0.015 ± 0.003 | 0.011 ± 0.002 | 0.010 ± 0.002 | **≤ 0.001** |
| **Diastolic Ca^2+^ concentration (nM)** | 213.9 ± 27.7 | 211.9 ± 33.4 | 231.7 ± 44.6 | 205.9 ± 30.6 | 259.5 ± 53.7 | 259.9 ± 29.7 | 290.6 ± 53.1 | 265.0 ± 40.5 | 255.4 ± 38.0 | 296.1 ± 51.0 | 285.7 ± 43.2 | 323.3 ± 53.5 | **0.03** |
| **Diastolic fluorescence (a.u.)** | 0.696 ± 0.020 | 0.692 ± 0.024 | 0.703 ± 0.028 | 0.689 ± 0.023 | 0.719 ± 0.032 | 0.728 ± 0.021 | 0.741 ± 0.034 | 0.728 ± 0.026 | 0.721 ± 0.024 | 0.744 ± 0.029 | 0.741 ± 0.025 | 0.761 ± 0.030 | **0.03** |
| **Systolic Ca^2+^ concentration (nM)** | 413.1 ± 51.9 | 466.6 ± 60.0 | 526.3 ± 88.2 | 478.5 ± 71.3 | 529.2 ± 96.3 | 471.8 ± 61.9 | 534.2 ± 86.5 | 522.6 ± 87.5 | 517.1 ± 97.0 | 551.5 ± 76.6 | 505.4 ± 112.2 | 498.4 ± 87.2 | 0.40 |
| **Systolic fluorescence (a.u.)** | 0.815 ± 0.025 | 0.840 ± 0.029 | 0.861 ± 0.032 | 0.842 ± 0.032 | 0.859 ± 0.035 | 0.842 ± 0.029 | 0.867 ± 0.037 | 0.859 ± 0.034 | 0.850 ± 0.036 | 0.872 ± 0.035 | 0.842 ± 0.038 | 0.845 ± 0.037 | 0.67 |
| **Ca^2+^ transient amplitude (a.u.)** | 0.119 ± 0.015 | 0.147 ± 0.016 | 0.159 ± 0.018 | 0.153 ± 0.020 | 0.140 ± 0.017 | 0.114 ± 0.015 | 0.125 ± 0.019 | 0.131 ± 0.021 | 0.129 ± 0.022 | 0.128 ± 0.023 | 0.101 ± 0.020 | 0.084 ± 0.016 | **0.04** |
| **Time to 50 % twitch relaxation (s)** | 0.091 ± 0.006 | 0.095 ± 0.005 | 0.089 ± 0.007 | 0.083 ± 0.006 | 0.070 ± 0.003 | 0.058 ± 0.002 | 0.094 ± 0.008 | 0.095 ± 0.011 | 0.097 ± 0.012 | 0.085 ± 0.011 | 0.074 ± 0.011 | 0.059 ± 0.002 | 0.50 |
| **Time to 90 % twitch relaxation (s)** | 0.275 ± 0.028 | 0.246 ± 0.017 | 0.227 ± 0.013 | 0.202 ± 0.015 | 0.160 ± 0.021 | 0.118 ± 0.012 | 0.330 ± 0.047 | 0.268 ± 0.041 | 0.231 ± 0.030 | 0.247 ± 0.036 | 0.180 ± 0.033 | 0.114 ± 0.009 | 0.14 |
| **Maximum rate of rise of stress (mN mm^-2^ ms^-1^)** | 0.221 ± 0.042 | 0.465 ± 0.111 | 0.776 ± 0.236 | 0.775 ± 0.182 | 0.592 ± 0.144 | 0.354 ± 0.067 | 0.190 ± 0.051 | 0.191 ± 0.036 | 0.284 ± 0.046 | 0.330 ± 0.070 | 0.223 ± 0.051 | 0.149 ± 0.036 | **≤ 0.001** |
| **Diastolic stress (mN mm^-2^)** | 2.00 ± 0.39 | 2.03 ± 0.31 | 2.07 ± 0.37 | 1.96 ± 0.43 | 2.26 ± 0.66 | 2.56 ± 0.74 | 2.42 ± 0.64 | 2.10 ± 0.63 | 2.34 ± 0.67 | 2.20 ± 0.56 | 2.85 ± 0.93 | 2.63 ± 0.71 | 0.87 |
| **Peak developed stress (mN mm^-2^)** | 9.11 ± 1.05 | 18.75 ± 3.85 | 25.17 ± 6.64 | 24.52 ± 4.99 | 19.07 ± 3.80 | 12.86 ± 2.00 | 8.58 ± 1.84 | 10.77 ± 2.42 | 11.79 ± 1.75 | 12.19 ± 2.12 | 9.53 ± 1.76 | 6.78 ± 1.24 | **≤ 0.001** |
| **Active stress (mN mm^-2^)** | 7.11 ± 1.24 | 16.72 ± 3.88 | 23.10 ± 6.65 | 22.56 ± 5.14 | 16.81 ± 3.98 | 10.29 ± 2.07 | 6.16 ± 1.75 | 8.66 ± 2.22 | 9.45 ± 1.59 | 10.00 ± 2.09 | 6.13 ± 1.48 | 4.15 ± 0.99 | **≤ 0.001** |

**Table 3. Force-frequency response Ca^2+^ and contractile measured parameters.**

Human RAA trabeculae Ca^2+^ handling and contractile measured parameters obtain over a range of stimulation frequencies. All experiments conducted at 37 °C in the presence of 1.5 mM Ca^2+^. All data is presented as mean ± SEM with significance determined by two-way ANOVA.
